# Tyrp1 Mutant Variants Associated with OCA3: Computational Characterization of Protein Stability and Ligand Binding

**DOI:** 10.3390/ijms221910203

**Published:** 2021-09-22

**Authors:** Milan H. Patel, Monika B. Dolinska, Yuri V. Sergeev

**Affiliations:** National Eye Institute, National Institutes of Health, Bethesda, MD 20892, USA; milanh.patel@hmhn.org (M.H.P.); dolinskam@nei.nih.gov (M.B.D.)

**Keywords:** melanogenesis, Tyrp1, OCA3, disease-related mutant variants, molecular modeling

## Abstract

Oculocutaneous albinism type 3 (OCA3) is an autosomal recessive disorder caused by mutations in the *TYRP1* gene. Tyrosinase-related protein 1 (Tyrp1) is involved in eumelanin synthesis, catalyzing the oxidation of 5,6-dihydroxyindole-2-carboxylic acid oxidase (DHICA) to 5,6-indolequinone-2-carboxylic acid (IQCA). Here, for the first time, four OCA3-causing mutations of Tyrp1, C30R, H215Y, D308N, and R326H, were investigated computationally to understand Tyrp1 protein stability and catalytic activity. Using the Tyrp1 crystal structure (PDB:5M8L), global mutagenesis was conducted to evaluate mutant protein stability. Consistent with the foldability parameter, C30R and H215Y should exhibit greater instability, and two other mutants, D308N and R326H, are expected to keep a native conformation. SDS-PAGE and Western blot analysis of the purified recombinant proteins confirmed that the foldability parameter correctly predicted the effect of mutations critical for protein stability. Further, the mutant variant structures were built and simulated for 100 ns to generate free energy landscapes and perform docking experiments. Free energy landscapes formed by Y362, N378, and T391 indicate that the binding clefts of C30R and H215Y mutants are larger than the wild-type Tyrp1. In docking simulations, the hydrogen bond and salt bridge interactions that stabilize DHICA in the active site remain similar among Tyrp1, D308N, and R326H. However, the strengths of these interactions and stability of the docked ligand may decrease proportionally to mutation severity due to the larger and less well-defined natures of the binding clefts in mutants. Mutational perturbations in mutants that are not unfolded may result in allosteric alterations to the active site, reducing the stability of protein-ligand interactions.

## 1. Introduction

Human tyrosinase-related protein 1 (Tyrp1) is a transmembrane, metal-containing glycoenzyme that catalyzes the oxidation of 5,6-dihydroxyindole-2-carboxylic acid (DHICA) to 5,6-quinone-2-carboxylic acid (IQCA). It is one of three tyrosinase-like enzymes in human melanocytes that are involved in the biosynthesis of melanin, a pigment found in hair, skin, and the iris of the eye. Mutations in the Tyrp1 gene (*TYRP1*) can lead to oculocutaneous albinism type 3 (OCA3), an autosomal recessive disease. Those with OCA3 typically present with one of two phenotypes: rufous OCA (ROCA) or brown OCA (BOCA). ROCA is characterized by red-bronze skin color, blue or brown irises, and ginger-red hair, while BOCA is characterized by light to brown hair and light to brown or tan skin color.

Four conserved regions exist among all three tyrosinase-like enzymes: an N-terminal signal peptide, an intra-melanosomal domain, one transmembrane α-helix, and a small C-terminal cytoplasmic domain. Within the intra-melanosomal domain, there exist both a Cys-rich subdomain and a tyrosinase-like subdomain containing a binuclear metal-ion binding motif. The crystal structure of the intra-melanosomal fragment of Tyrp1, which includes both subdomains (residues 25–470) was determined at 2.35 Å [1]. In total, Tyrp1 contains 537 residues comprising the signal peptide (1–24), the intra-melanosomal domain (Cys-rich subdomain 25–126, tyrosinase-like subdomain 127–477), the transmembrane α-helix (478–501), and the cytoplasmic domain (502–537).

**Figure 1:**
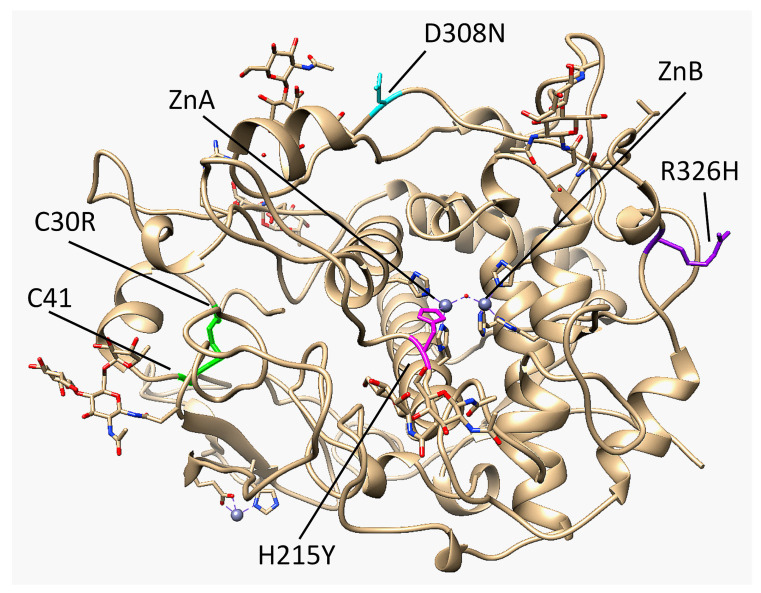
Location of each OCA3 mutant of interest in the Tyrp1 crystal structure (chain A of 5M8L). C30, C41, and the corresponding disulfide bridge are highlighted in green, H215 is highlighted in magenta, D308 is highlighted in cyan, R326 is highlighted in purple, and ZnA and ZnB are shown by grey spheres.

The core of the Cys-rich subdomain of Tyrp1 resembles the epidermal growth factor (EGF)-like domain due to its patterned disulfide bridges and short antiparallel ß-strands [1,2]. It contains five total disulfide bridges, three of them located within the EGF-like fold. Most albinism-related mutations of Tyrp1 affect its stability or activity, and expansion of the interface between the EGF-like domain and the tyrosinase-like domain was linked to OCA Type 1B mutations in tyrosinase [1,3]. One such mutation, C30R, breaks the C30-C41 disulfide bridge and results in OCA3 [4]. Tyrp1 was found to have a binuclear zinc active site rather than the typical binuclear copper active site found in tyrosinases (Figure 1). A four-helix bundle within the tyrosinase-like subdomain contains six total histidine residues that coordinate the Zn metal ions, which are bridged by a single water molecule. ZnA is coordinated by H192, H215, and H224, while ZnB is coordinated by H377, H381, and H404. In tyrosinase, histidine is essential for catalytic activity, and in both tyrosinase and Tyrp1, mutations to metal-coordinating histidine residues can result in reduced activity and consequently different forms of albinism [5,6]. Mutations to H215, H224, and H381 were specifically identified to have OCA3 [7,8]. Tyrp1 is an N-linked glycoprotein that contains sugars at six N sites (96, 104, 181, 304, 350, 385). Glycosylation has been shown to help ensure proper folding and stabilize tyrosinase-like structures during the process of translocation from the endoplasmic reticulum (ER) to the cytoplasm of the melanocyte [6,9,10].

The unfolding mutation screen (UMS) was developed to understand and evaluate the effect of missense mutations on protein folding and thermodynamic stability [11,12]. This program uses protein unfolding curves and thermodynamic changes in Gibbs free energy (ΔΔG), determined by FoldX to calculate propensities of mutations in global mutagenesis [13]. Initially, UMS [11] was tested with 16 crystal structures to evaluate the unfolding of 1391 mutations from the ProTherm database. UMS showed that the computational accuracy of the unfolding calculations was similar to the accuracy of previously published free energy changes, but provided a better scale. The results are then projected onto a protein model to highlight the critical residues and regions of structural importance to the protein [14]. 

While mutations leading to OCA3 have been discovered, the molecular mechanisms leading to these results have yet to be elucidated. Here, for the first time, we used computational methods to identify mechanisms behind mutation instability and differences behind protein-ligand interactions in Tyrp1 and OCA3-causing mutations. Understanding the differences in protein-ligand interactions among Tyrp1 and its associated mutations can establish mechanisms by which they change Tyrp1 catalytic activity. Coupled with *in vitro* and *in vivo* studies, in silico studies of similar nature can help lead to novel therapies for OCA3 patients.

## 2. Results

### 2.1. Crystal Structure of Tyrp1 and OCA3-Related Mutants

Tyrp1 is a metalloenzyme involved in melanin synthesis. Mutations to the *TYRP1* gene can result in OCA3. The location of four OCA3 mutations was analyzed using the crystal structure of Tyrp1 (Figure 1). C30 is located within the Cys-rich subdomain of human Tyrp1 and forms the first of five disulfide bridges with C41. A mutation from cysteine to arginine (C30R) breaks the disulfide bridge. Between C30 and C41 in Tyrp1 exists a loop with a small α-helix. The Cys-rich subdomain is a very stable region of Tyrp1 so removing a disulfide bridge could result in a loss of stability and greater flexibility within the region. H215 is one of the six zinc-coordinating histidine residues, coordinating ZnA. Mutating histidine to tyrosine (H215Y) removes a coordination bond for ZnA, leaving it with only two coordination sites. This mutation could change how the active site is folded while the protein is synthesized in the ER, affecting its ability to catalyze the reaction from DHICA to IQCA. D308 is located on the periphery of Tyrp1. The mutation from aspartic acid to asparagine (D308N) could introduce the formation of two transient hydrogen bonds with the nearby D291 (Supplementary Figure S1). R326H is also located on the periphery of Tyrp1. The mutation from arginine to histidine (R326H) removes the two transient hydrogen bonds between R326 and E329, resulting in a loss of stability (Supplementary Figure S2). 

OCA3-related mutant variants were analyzed computationally to understand their protein stability. Protein residue-residue distance maps were created in search of structural changes, which were caused by molecular dynamic simulations. In addition, we looked for the most stable conformations of the mutant binding sites and docking Tyrp1 ligands to understand a possible mechanism of Tyrp1 catalytic activity.

### 2.2. Mutant Variant Stability

To determine the severity of the thermodynamic changes in each OCA3 mutation in comparison to Tyrp1, the Tyrp1 structure was run through the Unfolding Mutation Screen (UMS) [11,12]. UMS ascribed each mutation a ∆∆G value and unfolding parameter to describe the thermodynamic change in free energy and the fraction of unfolded protein, respectively (Table 1). 

Each Tyrp1 residue also received a foldability parameter or an aggregated sum of the propensities of the unfolding parameters of that residue’s mutation. The foldability parameter is meant to highlight the most critical residues involved in the structural and thermodynamic stability of the protein [11,15]. C30 and H215 are expected to contribute the most to protein stability with foldability values of 18.99 and 19.85, respectively. These values are consistent with the unfolding parameters of the C30R and H215Y mutations, which are both 1.00, indicating a complete unfolding or misfolding of the mutant. D308 and R326 are deemed to be less integral to Tyrp1 stability per UMS with foldability parameters of 0.98 and 6.74, respectively. The D308N and R326H mutants reflect the foldability parameters with unfolding parameters of 0.56 and 0.98. The mutants are expected to be stable molecules with the native protein fold preserved. The predicted effect of the mutation was matching the available ClinVar data (ClinVar (nih.gov)) for H215Y and R326H. The effect of mutation C30R was expected to be pathogenic [4]. 

**Figure 2:**
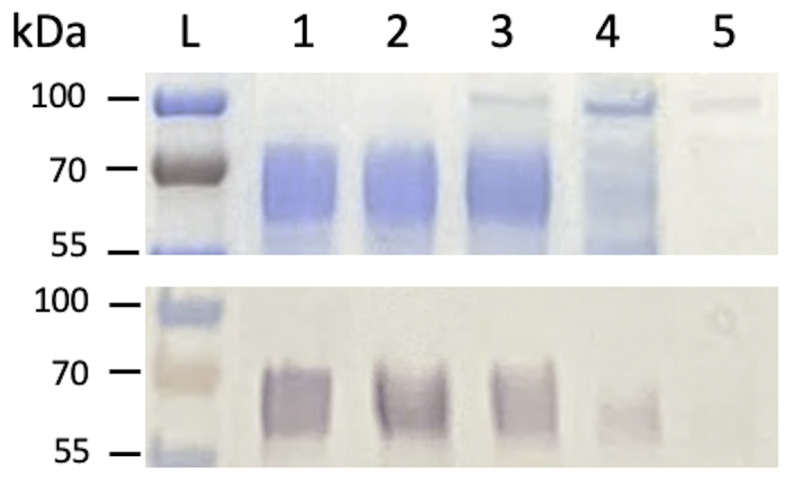
SDS-PAGE and Western blot of Tyrp1 and OCA3-related mutants. SDS-PAGE (top panel) and Western blot (bottom panel) showing the expression of Tyrp1 and OCA3-related mutants after purification by IMAC and GF chromatography. From the left: L, protein ladder; Tyrp1; 2, D308N; 3, R326H; 4, H215Y, and 5, C30R.

The results of in silico analysis of protein stability were verified experimentally and will be presented in the accompanying experimental manuscript (Dolinska et al., in preparation). Briefly, to investigates the effect of genetic mutations on the human Tyrp1 structure, function, and stability, we expressed and purified the intra-melanosomal domain of Tyrp1 located in residues 25–537 of the native protein and its OCA3-related mutants, C30R, H215Y, D308N, and R326H. The proteins were produced in the whole insect *Trichoplusia ni* larvae, purified by immobilized metal affinity (IMAC) and gel filtration (GF) chromatography, and analyzed using the SDS-PAGE gel and Western blot. Tyrp1, D308N, and R326H migrated through the SDS-PAGE gel as broad, heterogeneous bands between 55 and 70 kDa (Figure 2, upper panel). These bands reacted strongly with the anti-Tyrp1 antibody (TA99, Santa Cruz Biotechnology) (Figure 2, low panel). The heterogeneity of the protein’s molecular weight can be attributed to the N-linked glycosylation of the enzymes. In contrast, C30R and H215Y mutants indicate no (or very weak) bands at the proper position on SDS-PAGE gel and the Western blot. Both mutants are not properly folded and are therefore unstable and aggregate during the purification process. Our experimental data confirmed that the foldability analysis could correctly evaluate the effect of mutations critical for protein stability.

Finally, we demonstrated by using both UMS and experimental SDS-PAGE that the C30R and H215Y mutants are not stable due to severe damage from genetic perturbation. We expect that two other mutant proteins, D308N and R326H, are stable and will show some catalytic activity.

### 2.3. Cys-rich Subdomain Instability

Mutant variants were subjected to molecular dynamics (MD) as described in Section 5 and were further investigated using residue-residue distance maps, which were generated for all five structures in UCSF Chimera [16] (Supplementary Figure S3). Each structure’s map compared distances across five timestamps at 0, 25, 50, 75, and 100 ns to provide a comprehensive characterization of the MD simulation.

**Figure 3:**
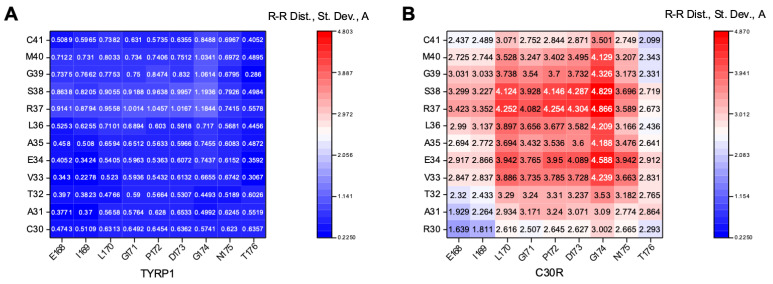
A portion of residue-residue distance maps of the Tyrp1 (**A**) and the C30R (**B**) structures across five timestamps: 0, 25, 50, 75, and 100 ns. Maps display the standard deviation of distances between residues. The C30-C41 region in Tyrp1 and R30-C-41 region in C30R were compared with a neighboring loop (E168-T176) to highlight the flexibility introduced by the C30R mutant within the Cys-rich subdomain. C30R shows a greater degree of fluctuation (maximum standard deviation of 4.87 Å) than Tyrp1 (maximum standard deviation of 1.19 Å).

**Figure 4:**
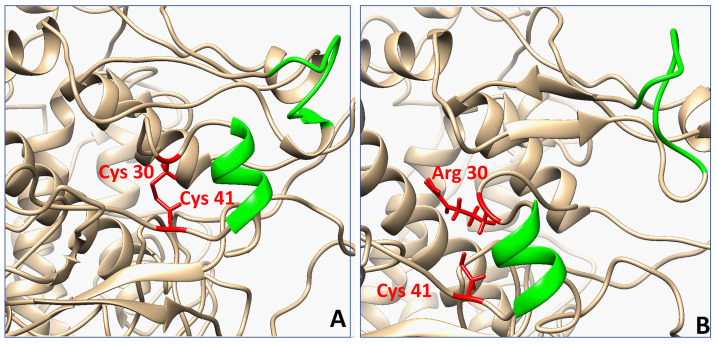
Compared region of residue-residue distance maps in Tyrp1 and C30R mutant. Homology models of Tyrp1 (**A**) and C30R (**B**) were equilibrated using MD in water for 100 ns. Residues in red indicate the intact and the broken (C30R) disulfide bridge. The residues in red and green indicate the focused region of the residue-residue distance maps in Figure 2.

The most interesting case was the C30R mutant, which broke the C30-C41 disulfide bridge. UMS expects C30 mutants to have severe effects on Tyrp1 structural and thermodynamic stability, and the C30R mutant specifically is expected to have a complete loss of protein stability (Table 1). The expression and purification of C30R are consistent with the values generated by UMS. Consequently, the mutant contained notable fluctuations in the R30-C41 region. To highlight the effect of these fluctuations, a small portion of the map is shown in Figure 3. C30-C41 is one of five disulfide bridges within the Cys-rich subdomain, and in between the two cysteine residues is a short α-helix (V33-S38) (Figure 4A). The C30-C41 region (R30-C41 in the C30R mutant) was mapped against the E168-T176 region, a neighboring loop (Figure 4B). C30R demonstrated a much higher level of flexibility within this region with a standard deviation of distances up to 4.87 Å, compared to 1.19 Å in Tyrp1. No other mutant had a standard deviation above 1.27Å in the region (Supplementary Figure S3). The C30-C41 region is located within the Cys-rich subdomain, indicating an introduction of flexibility due to the loss of the C30-C41 disulfide bridge in the C30R mutant. 

### 2.4. Free Energy Landscapes

**Figure 5:**
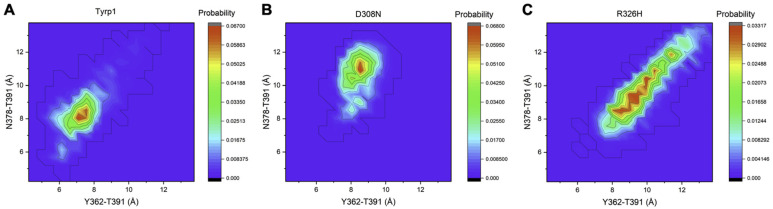
Free energy landscapes of Tyrp1 (**A**), D308N (**B**), and R326H (**C**). The peak of Tyrp1 was centered around (7.10, 8.05) Å with a probability of 0.067. The peak of D308N was centered around (8.52, 10.90) Å with a probability of 0.068. The peak of R326H was centered around (8.52, 8.52) Å with a probability of 0.032. The binding sites of Tyrp1 and D308N appear to be better defined than R326H’s binding site.

Free energy landscapes are usually obtained to investigate the relative stabilities of different conformational states in a biomolecule concerning a conformation coordinate of interest [17]. In our work, free energy landscapes were created for Tyrp1 and two mutant variants, D308N and R326H, which are expected to form stable protein structures. A macro was created in YASARA to measure the distance between T391 and both Y362 and N378 for every simulation snapshot (Table 2). T391, Y362, and N378 are the residues expected to form the bottleneck for the passage of the DHICA molecule (Radius = 3.45 Å). Tyrp1 had a mean of 7.37 Å with a standard deviation of 1.05 Å for the Y362-T391 distance and a mean of 8.47 Å with a standard deviation of 1.28 Å for the N378-T391 distance. D308N had a mean of 8.45 Å with a standard deviation of 0.65 Å for the Y362-T391 distance and a mean of 10.62 Å with a standard deviation of 1.65 Å for the N378-T391 distance. R326H had a mean of 9.90 Å with a standard deviation of 1.50 Å for the Y362-T391 distance and a mean of 9.93 Å with a standard deviation of 1.65 Å for the N378-T391 distance. Distances were inputted into the Weighted Histogram Analysis Method (WHAM) to generate free energy landscapes for all three structures [18,19,20]. The free energy of each bin of data was related to probability using the Boltzmann distribution to identify the most probable conformations of the bottleneck residues. Tyrp1 and D308N appeared to be well-defined peaks centered around the (7.10, 8.05) Å with a probability of 0.067 and (8.52, 10.90) Å with a probability of 0.068, respectively (Figure 5A,B). The D308N landscape indicates that the most probable distances between the bottleneck suggest a larger opening to the active site (Figure 5B). R326H is a much less well-defined contour, with no central peak (Figure 5C). The most probable data bin is centered around (8.52, 8.52) with a probability of 0.032. Overall, Tyrp1 has a more compact bottleneck than the bottlenecks of both D308N and R326H, potentially allowing for tighter binding and stronger interactions with DHICA. As a result, both mutant variants are expected to have reduced catalytic activity.

### 2.5. Ligand–Receptor Interactions

The prediction of the reduced catalytic activity of mutant variants was further confirmed from the analysis of ligand-receptor interactions. DHICA was docked to the most probable bottleneck conformations of Tyrp1, D308N, and R326H per WHAM.

**Figure 6:**
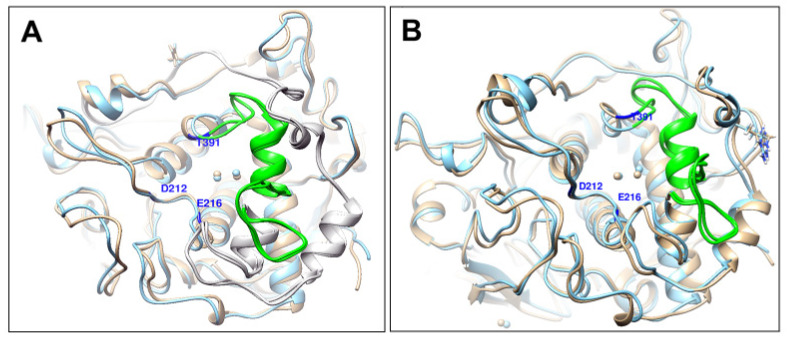
Changes in a mutant protein structure are caused by genetic perturbations. Mutant variants, D308N (**A**) and R326H (**B**), and Tyrp1 superposition demonstrates the changes in a whole mutant protein structure. Mutant protein and Table 1. ribbons are shown in blue and gold, respectively. The green part of the ribbon corresponds to residues 362–391.

**Figure 7:**
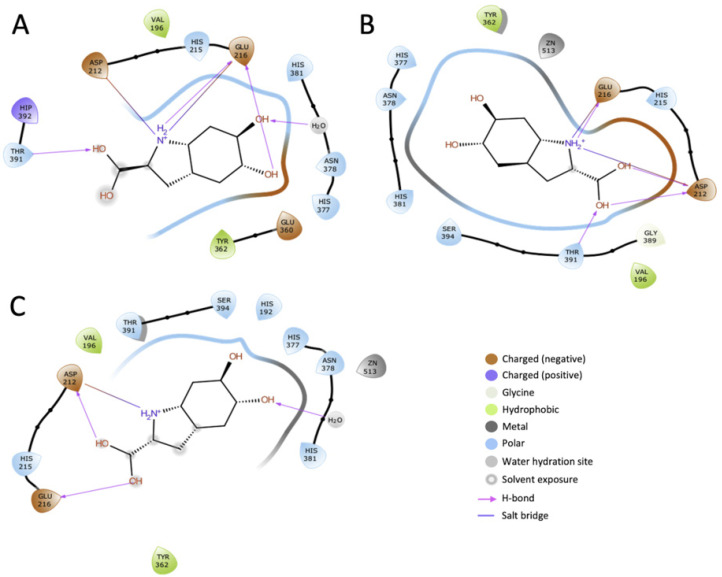
Docked DHICA interaction with Tyrp1 (**A**), D308N (**B**), and R326H (**C**). Tyrp1 and D308N have six total interactions with DHICA: four hydrogen bonds and two salt bridges. R326H has two fewer interactions with DHICA: three hydrogen bonds and one salt bridge.

**Figure 8:**
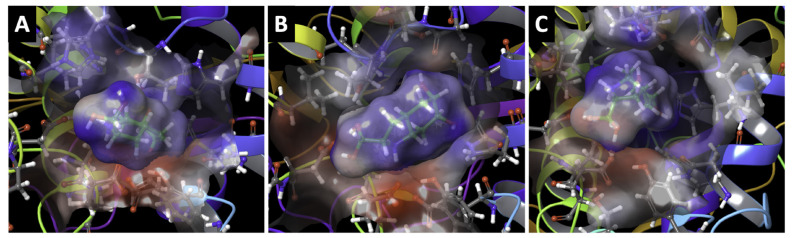
Molecular docking of DHICA to Tyrp1 (**A**), D308N (**B**), and R326H (**C**). Each docking result has DHICA within two water molecules of zinc with the hydroxyl groups on the aromatic ring oriented towards the active site. Structural images indicate that the partial positive of the NH_2_ group of DHICA is oriented between the partial negative COO^−^ side chains of D212 and E216. Docking experiments suggest as the severity of the mutation increases, DHICA is less tightly bound to the active site.

The effect of mutations D308N and R326H is shown in Figure 6A,B, respectively. Ligand–receptor interactions are detailed in Figure 7A–C. Docking results were considered only if the hydroxyl groups on the aromatic ring of DHICA were oriented towards the active site and were within two water molecules of zinc. Interaction-related distances are listed in Table 3. The results indicate that the partial positive of the NH_2_ functional group of DHICA orients itself between the partial negative COO^−^ side chains of D212 and E216. Both Tyrp1 and D308N have six total interactions with DHICA: four hydrogen bonds and two salt bridges. In Tyrp1, E216 accepts two hydrogen bonds and forms one salt bridge, D212 forms one salt bridge, and both T391 and the zinc-bridging water molecule donate one hydrogen bond to DHICA. One such hydrogen bond is donated from the zinc-bridging water molecule, indicating that the water may serve multiple functions within the active site. The distance from DHICA to a Tyrp1 zinc atom is 4.15 Å. The salt bridge lengths are 2.79 Å and 3.91 Å for D212 and E216, respectively. In D308N, D212 accepts two hydrogen bonds and forms one salt bridge, D212 accepts one hydrogen bond and forms one salt bridge, and T391 donates one hydrogen bond. The distance from DHICA to a D308N zinc atom is 4.51 Å. The salt bridge lengths are 4.31 Å and 4.49 Å for D212 and E216, respectively. R326H has four total interactions with DHICA: three hydrogen bonds and one salt bridge. The distance from DHICA to an R326H zinc atom is 3.63Å. E212 accepts one hydrogen bond and forms one salt bridge, D216 accepts one hydrogen bond, and the zinc-bridging water molecule donates one hydrogen bond. Structural images indicate that a more open bottleneck affects the ligand-receptor interactions in the D308N and R326H mutants (Figure 8). Gaps begin to appear between the surfaces of the ligand and the active site in D308N and are exacerbated in R326H, demonstrating a weaker active site-bound ligand. R326H-DHICA interactions appear to only have one salt bridge due to the larger opening in the active site. As the oxidation of DHICA occurs, the two salt bridges seem to be integral to ligand stabilization within the active site. In comparison with Tyrp1, the weakened and fewer interactions with DHICA in D308N and R326H may result in a less stable protein-ligand complex, affecting the oxidation from DHICA to IQCA. The weakening of protein-ligand binding might indicate the reduced catalytic activity of both mutant variants.

## 3. Discussion

Tyrp1 is one of three key enzymes involved in the melanogenesis pathway, which results in the production of melanin pigment. It is a glycoenzyme that catalyzes the oxidation of DHICA to IQCA. Mutations to the *TYRP1* gene can result in the autosomal recessive disorder OCA3. Four OCA3 mutants of interest were chosen from Human Gene Mutation Database (HGMD) Professional | QIAGEN to better understand Tyrp1 protein stability and catalytic activity: C30R, H215Y, D308N, and R326H. Global mutagenesis and Western Blot suggested that C30R and H215Y were severely unstable, while D308N and R326H conserved some level of catalytic activity. Free energy landscapes of the Tyrp1 bottleneck demonstrated that a slight loss of catalytic activity in D308N and R326H may be due to a larger active site, affecting interactions that stabilize DHICA during catalysis. These non-covalent interactions include salt bridges from D212 and E216 as well as various hydrogen bonds. Structural perturbations resulting from the D308N and R326H mutants may result in allosteric alterations to the active site that affect the stability of the ligand during the redox reaction.

UMS predicted mutants C30R and H215Y would be completely misfolded or unfolded, and mutants D308N and R326H to retain some stability. The results of global mutagenesis were confirmed by experimental SDS-PAGE and Western Blot, providing further validity to UMS and its ability to predict mutant stability using the foldability parameter.

C30R is in the Cys-rich subdomain, and it breaks the C30-C41 disulfide bridge. The Cys-rich subdomain of Tyrp1 is an extremely stable portion of the protein and a loss of a disulfide bridge may introduce thermodynamic instability and structural flexibility to this region. While the simulation did not suggest mutant unfolding, the mutation could alter the folding process while C30R is synthesized in the ER, resulting in a completely misfolded or unfolded protein.

H215Y is located within the active site, and it removes one of three coordination sites for ZnA. UMS also expects mutations of H215 to have a severe effect on the structural and thermodynamic stability of Tyrp1, and the ∆∆G value of H215Y indicates the greatest effect out of all four mutants of interest. The expression and purification of H215Y are consistent with the results generated by UMS. The loss of a coordination bond is expected to affect active site stability, particularly the zinc atom’s ability to remain situated within the binding pocket, resulting in a lack of catalytic activity. A comparison of H215Y structures at various points in the simulation did not suggest any active site structural alterations. All four transmembrane helices and the zinc-water-zinc complex remained in place. Residue-residue distance maps did not suggest structural fluctuations not seen in Tyrp1. However, since the mutant is expected to be unfolded or misfolded, the lack of trends towards structural alterations indicates that the protein may be unable to properly complete the folding process as it is synthesized in the ER. Similar results were seen in copper-coordinating histidine OCA1A mutations in human tyrosinase (Tyr) [3]. It was suggested that the folding of Tyr occurs in four distinct stages. Folding of the Cys-rich region and folding of each half of the transmembrane helix bundle comprise the first three stages, with the latter two stages forming the active site. Similar to Tyr, the Tyrp1 helix bundle is comprised of two helix-loop-helix motifs. Each motif contains three zinc-coordinating histidine residues and is small enough to form within a ribosome exit tunnel. Once the entire protein is processed by the ribosome, both helix-loop-helix motifs come together with the zinc-water-zinc complex to form the active site. However, without all six zinc-coordinating histidine residues, the zinc atoms are unable to correctly position themselves within the active site, and the transmembrane helix bundle does not form, leading to a full loss of catalytic activity in Tyrp1 due to denaturation.

D308N is located on the periphery of the protein, and it introduces the formation of two transient hydrogen bonds. D308 is not expected to be critical to Tyrp1 stability, and UMS does not expect D308N to be unfolded or misfolded, having the lowest ∆∆G of any of the four mutants. The expression and purification of D308N are consistent with UMS results. The addition of hydrogen bonds suggests that the stability of the mutant region might be increased. Effects of the mutation may result in allosteric alterations to active site structure as described by the most probable conformations of the bottleneck residues. The size of the bottleneck increases compared to Tyrp1, and computational docking experiments indicate that this difference may affect ligand-receptor interactions. While the number and type of noncovalent interactions between DHICA and D308N remain similar to those of Tyrp1, the strength of the stabilizing salt bridges decreases due to distance. The increased distances of these interactions are reflected in the surface images, where DHICA is not as tightly bound to the active site, leading to a loss of catalytic activity in D308N.

R326H is also located on the periphery of the protein, and it has two transient hydrogen bonds with E329. Mutations to R326 are expected to have varying levels of influence on Tyrp1 stability, and UMS does not expect R326H to be completely unfolded or misfolded. The expression and purification of R326H are consistent with UMS results. The removal of two hydrogen bonds suggests some loss of stability, and like D308N, this change in protein stability may result in allosteric alterations to the bottleneck structure. Again, the bottleneck increases compared to Tyrp1′s bottlenecks, but unlike D308N, the free energy landscapes do not indicate any centralized peak, and the altered bottleneck affects ligand-receptor interactions. The total number of noncovalent interactions decreases, subsequently decreasing the stability of the ligand in the active site. Fewer interactions are reflected in the surface images, where large gaps appear between DHICA and R326H. These gaps indicate the DHICA is not as tightly bound to the protein, leading to a loss of catalytic activity in R326H.

While both the D308N and R326H mutations do not directly affect the stability of the active site, their allosteric impacts may result in free energy changes. Enzymes have been shown to have residues that are part of allosteric regulatory sites critical to long-range communication [21]. A conservative replacement of D308 and R326 could result in free energy alterations that affect protein-ligand interactions. Both mutations are within 15 Å of transmembrane helices containing zinc-coordinating histidine residues, and mutational perturbations could affect the packing and dynamics of these helices that comprise the active site. Previous studies have shown that this long-range modulation of protein features can alter protein stability, thereby contributing to allosteric modulation of function [22]. Both D308 and R326 may be part of an intra-protein interaction network that allosterically affects the structure of the active site, particularly the bottleneck residues. This allosteric effect could be interpreted at the level of interatomic interactions. The addition and removal of hydrogen bonds in D308N and R326H, respectively, could perturb the transmembrane helices, increasing the size of the bottleneck and weakening the interactions between DHICA and the protein. These weakened interactions, in turn, affect the stabilization of DHICA within the active site and could result in reduced catalytic activity. In both the Tyrp1 and R326H docking experiments, DHICA was shown to accept a hydrogen bond from the active site water molecule. While previous studies indicated that the water molecule’s purpose was to bridge the zinc atoms, it may serve multiple functions within the active site, including stabilization of the DHICA ligand.

Although UMS foldability has shown to be an accurate predictor of protein expression and stability, it remains to be seen if this translates to phenotypic variations in OCA3. Since D308N and R326H are expected to have some level of protein activity, patients with these mutations should have reduced the severity of OCA3. If UMS can accurately predict the extent of these mutations, it could be a useful resource in helping guide personal patient therapies to those affected by OCA3.

## 4. Methods

### 4.1. Molecular Modeling and Simulation

Four OCA3 mutations (C30R, H215Y, D308N, and R326H) were selected from HGMD [4,7,23]. They were generated using the Edit > Swap > Residue function on the 5M8L PDB file in YASARA (http://www.yasara.org/, accessed on 15 July 2021). All four mutants and the original 5M8L structure were subjected to 100 ns of MD using YASARA’s ‘run.mcr’ macro. Ion concentration was added as a mass fraction with 0.9% NaCl. The simulation temperature was set to 298 K with a water density of 0.997 g/mL. Tyrosinases are synthesized in the ER and function in melanosomes at pH 5.5. To replicate the melanosome environment, the simulation pH was set to 5.5. The cell size extended to 10 Å beyond each side of the protein in the shape of a cube with dimensions 92.9 Å × 92.9 Å × 92.9 Å. Each simulation was run in YASARA using an AMBER14 forcefield, with a timestep of 2.5 fs. Simulation snapshots were outputted for every 0.1 ns, resulting in 1000 simfiles for each simulation.

### 4.2. Unfolding Mutation Screen

Global mutagenesis was conducted on Tyrp1, and each mutant was characterized by a thermodynamic change in Gibbs free energy (ΔΔG) [11,12]. These values were calculated using the semi-empirical method (FoldX) and standardized on a 0–1 scale as the unfolding parameter, or the fraction of protein in the unfolded state [11,13]. UMS also outputted a foldability parameter to show critical residues in protein folding. This parameter is a sum of severity-weighted unfolding propensities for the 19 mutations generated at a specific residue. Residues with the highest foldability were considered critical for protein folding [11]. The full-atomic TYRP1 homology model and the results of the unfolding mutation screen are freely available from the ocular proteome website at the NEI Commons (https://neicommons.nei.nih.gov/#/proteomeData/, accessed on 19 July 2021).

### 4.3. Free Energy Landscape

Free energy landscapes were created for the Tyrp1, D308N, and R326H to determine the highest probability conformation of the binding site. The active site bottleneck residues were determined to be Y362, N378, and T391 (Supplementary Figure S4). A weighted histogram analysis method was used to determine the free energy [18,19,20]. The 2D version was used so that two reaction coordinates comprising the three residues would be considered in the free energy landscape. Two distances Y362(OH)-T391(OG1) and N378(OD1)-T391(OG1) were chosen as non-periodic reaction coordinates. A macro was created and run in YASARA to calculate the distances at every simulation snapshot, resulting in a dataset of 1000 points. The data points were sorted into 21 bins with a minimum bin of 4 and a maximum bin of 14. WHAM parameters included 10^−5^ for the tolerance, 298 K for the temperature, 0 for the number of padding values (due to the aperiodic reaction coordinate), and 0 for the spring constant.

### 4.4. Docking Experiments

PDB files were created for Tyrp1, D308N, and R326H at timestamps where the Y362-T391 and N378-T391 distances corresponded to the highest probability bin per WHAM. The protein structure was imported, processed, and refined at pH 5.5 using the Protein Preparation Wizard tool in Maestro [24]. A preprocessing job was submitted with assigned bond orders, use of CCD database, add hydrogens, create zero bond orders, create disulfide bonds, and delete waters beyond 5.00 Å from heteroatom groups turned on in pH 5.5. Hydrogen bonds were optimized with water sampling on, and crystal symmetry and minimize hydrogens of altered species off in pH 5.5. Restrained minimization was conducted using an RMSD of 0.3 in forcefield OPLS3e. A 2D structure of DHICA was created in Open Babel [25]. Then, 32 DHICA states were generated using the LigPrep function. The states were generated in Epik in pH 5.5 using an OPLS3e forcefield and retaining specified chiralities, with desalting and generating tautomers on [26,27]. A receptor grid was created using the Receptor Grid Generation function. The receptor box was centered around the two zinc atoms and the water molecule, and the box was set to 10 Å within the centroid. The Van der Waals (VDW) radius scaling factor was set to 1.0, and the partial charge cutoff was 0.25. No constraints were added. All 32 DHICA states were docked to the generated receptor grid using the Ligand Docking function. The VDW scaling factor was 0.8 with a partial charge cutoff of 0.15. Standard precision and flexible ligand sampling were used with sample nitrogen inversions, sample ring conformations, bias sampling of torsions for all predefined functional groups, and add Epik state penalties to docking score turned on. No constraints were added. Docking results were only considered if the hydroxyl groups of DHICA docked within two water molecules of the binuclear zinc active site (~5.5 Å).

### 4.5. Structure Comparison

Tyrp1 and OCA3 mutant structures were compared in UCSF Chimera [16]. Residue-residue distance maps were created to highlight where the movement occurred over time. PDB files were created at 0, 25, 50, 75, and 100 ns of the simulation for all five structures. The structures were aligned using the MatchMaker tool, with the structure as 0 ns used as a reference. The ‘best aligning pair of chains between reference and match structures’ option was selected for chain pairing, Needleman–Wunsch was selected as the alignment algorithm, BLOSUM-62 was selected as the matrix, with a gap extension of 1 and include secondary structure score of 30% and compute secondary structure assignment turned on. Matching was conducted by iterating by pruning along atom pairs until no pair exceeds 2.0 Å. Residue-residue distance maps were created for all five structures using the RR Distance Maps function by importing the five timestamped files for each structure. Both the average distance and standard deviation were calculated for each map.

### 4.6. In Vitro Analysis of Tyrp1 and OCA3-Related Mutants

Recombinant human wild-type intra-melanosomal domain of the tyrosinase-related protein (residues 25–537 of the native protein), Tyrp1, and OCA3-related mutants, C30R, H215Y, D308N, and R326H, were expressed in baculovirus and produced in whole insect *T. ni* larvae. Proteins were purified using methods previously described [28,29], and analyzed by SDS-PAGE using 4–15% polyacrylamide gels (Bio-Rad, Hercules, CA, USA) stained with Thermo Scientific GelCode Blue reagent (Thermo Scientific, Schaumburg, IL, USA). Protein identity was confirmed by Western blot analysis using an anti-Tyrp1 antibody (TA99, Santa Cruz Biotechnology, Santa Cruz, CA, USA, 1:500 dilution).

## 5. Conclusions

For the first time, the mutant variants of Tyrp1, C30R, H215Y, D308N, and R326H were analyzed computationally. We have demonstrated that the protein stability changes caused by the disease-causing mutation could be predicted using the unfolding mutation screen. For three mutant variants, the predicted changes are associated with OCA3 clinical data on genetic disease severity. We demonstrated that both the C30R and H215Y mutants result in a complete loss of protein stability as they affect structurally integral regions of Tyrp1, which is consistent with *in vitro* experiments. Both D308N and R326H conserve the structure of these regions but may allosterically affect the conformation of the active site, increasing the size of the bottleneck. This effect is highlighted in protein-ligand interactions. While the noncovalent interactions largely remain similar between Tyrp1 and D308N, their strength decreases due to distance. R326H, however, is shown to have an even greater effect on the bottleneck, resulting in both fewer and weaker interactions with DHICA. Identifying the bottleneck residues of active sites can help identify their most stable conformations and elucidate protein-ligand interactions integral to catalytic activity. These studies, coupled with *in vitro* and *in vivo* experiments, can advance our understanding of the mechanisms behind mutations of Tyrp1 and lead to novel therapies for patients with OCA3.

## Figures and Tables

**Table 1:** Foldability, unfolding parameters, and ∆∆G values generated by UMS for each residue and mutation of interest.

Residue	Foldability Parameter	OCA3 Mutation	Unfolding Parameter	∆∆G	Tyrp1 Polypeptide from SDS-PAGE	Clinical Significance (ClinVar)
C30	18.99	C30R	1.00	16.21	No band	No melanin^2^
H215	19.85	H215Y	1.00	17.82	No band	Pathogenic
D308	0.98	D308N	0.56	0.14	~60 kDa	Unknown
R326	6.74	R326H	0.98	2.31	~60 kDa	Benign

**Table 2:** Bottleneck distance results.

	Mean (Å)	Std. Dev. (Å)	Min. (Å)	Max (Å)
Y362-T391				
Tyrp1	7.37	1.05	5.02	11.89
D308N	8.45	0.65	6.70	10.35
R326H	9.90	1.50	6.17	14.30
N378-T391				
Tyrp1	8.47	1.28	4.83	13.62
D308N	10.62	1.11	6.93	12.94
R326H	9.93	1.65	6.03	14.51

**Table 3:** Ligand–receptor interaction-related distances.

	**Tyrp1**	**D308N**	**R326H**
Distance to Zinc	4.15	4.51	3.63
Salt Bridge Length D212 (Å)	2.79	4.31	3.93
Salt Bridge Length E216 (Å)	3.91	4.49	n/a

## Data Availability

Homology model and global mutagenesis data for Tyrp1 are freely available at NEI Commons website (https://neicommons.nei.nih.gov/#/proteomeData, accessed on 18 July 2021).

## Supplementary Materials

The following are available online at www.mdpi.com/article/10.3390/ijms221910203/s1, Supplementary Figure S1: OCA3-related mutation D308R, Supplementary Figure S2: OCA3-related mutation R326H, Supplementary Figure S3: Residue-Residue distance maps, Supplementary Figure S4: Bottleneck residues of Tyrp1.
