# Supplementary material for: Tyrp1 Mutant Variants Associated with OCA3: Computational Characterization of Protein Stability and Ligand Binding
Source: Int J Mol Sci. 2021 Sep 22;22(19):10203. doi: 10.3390/ijms221910203 (PMC8508144; doi:10.3390/ijms221910203)
Supplement: Supplementary file 1 [file ijms-22-10203-s001.zip › ijms-1363929-supplementary.pdf]

Supplementary Figures

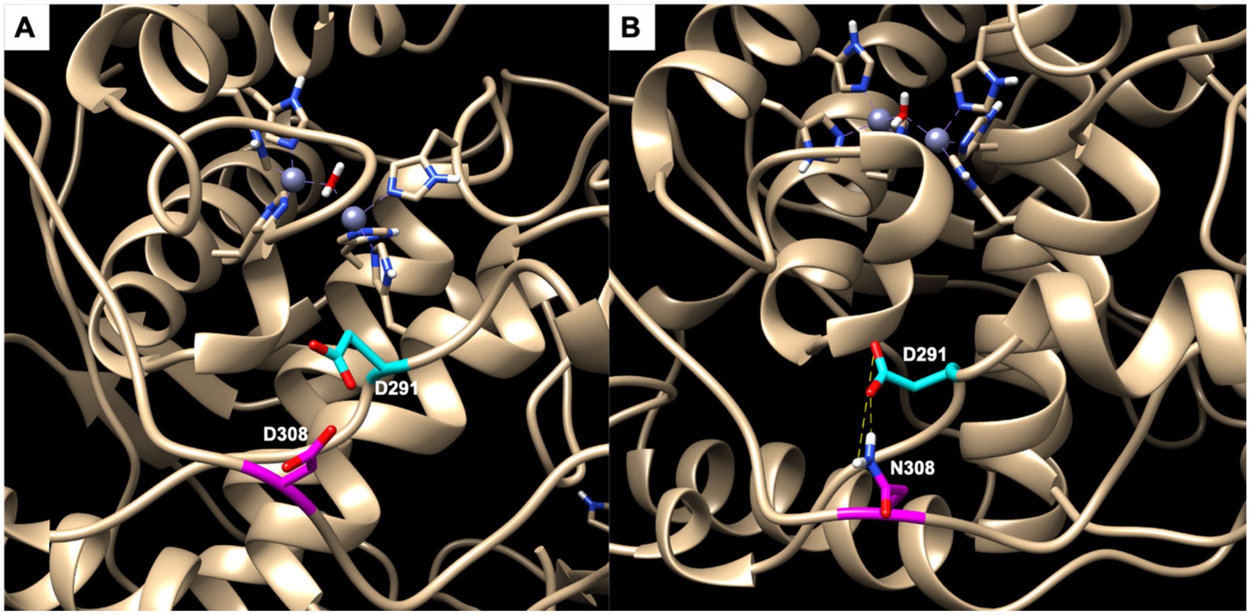

Supplementary Figure S1. Environment for D308 (A) and N308 (B). The mutation from Asp to Asn introduces two potential hydrogen bonds between the side chains of R308 and D291. Mutational perturbations could affect the nearby transmembrane helix containing H192, a copper-coordinating His residue.

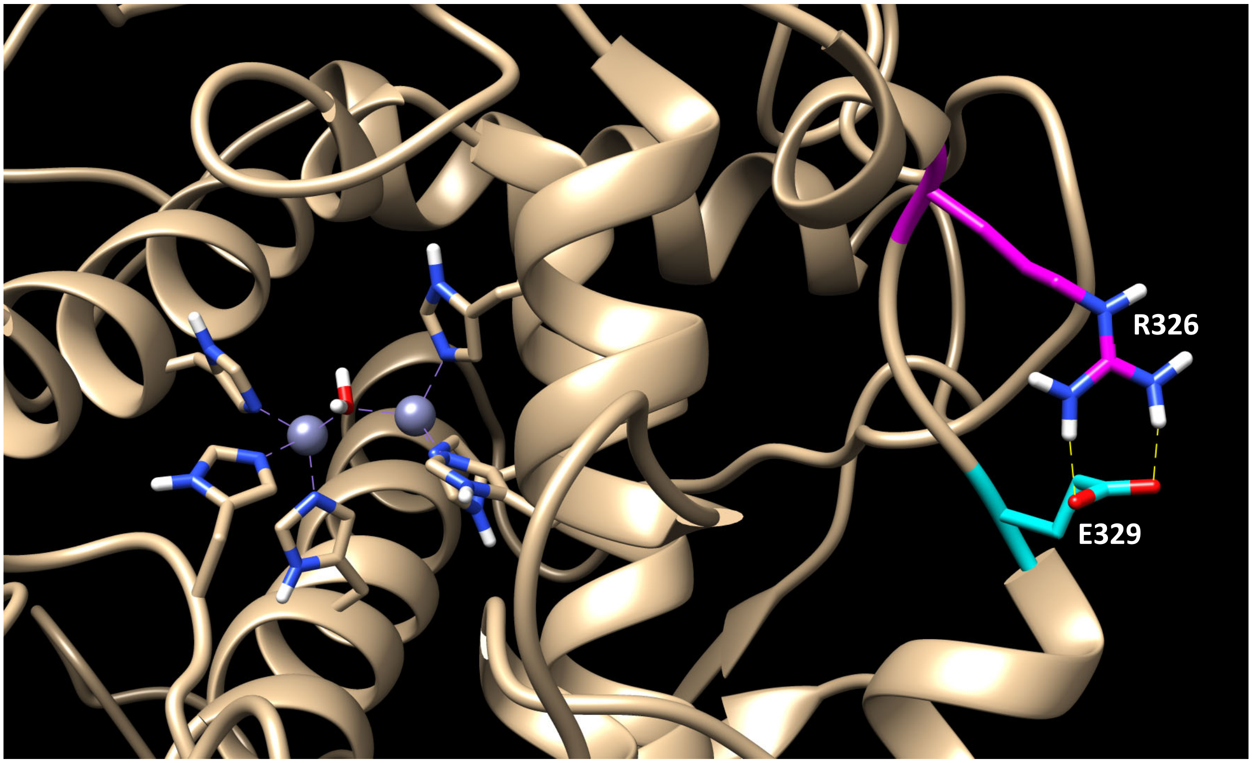

Supplementary Figure S2. Environment of R326. R326 forms two transient hydrogen bonds with E329. A mutation from Arg to His removes both hydrogen bonds, due to the shorter length of the His side chain. Mutational perturbations could affect the nearby transmembrane helix containing H377 and H381, two copper-coordinating His residues.

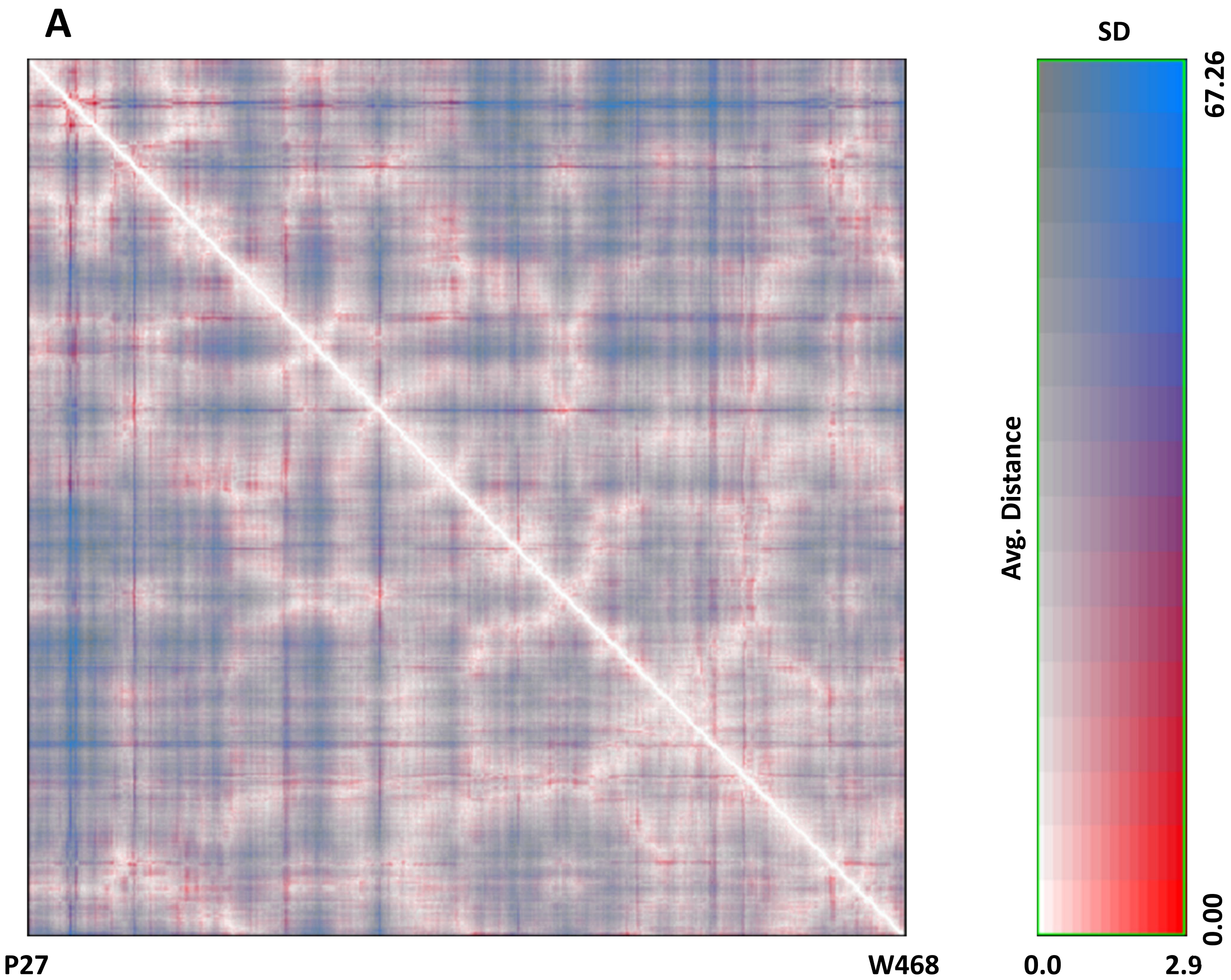

B

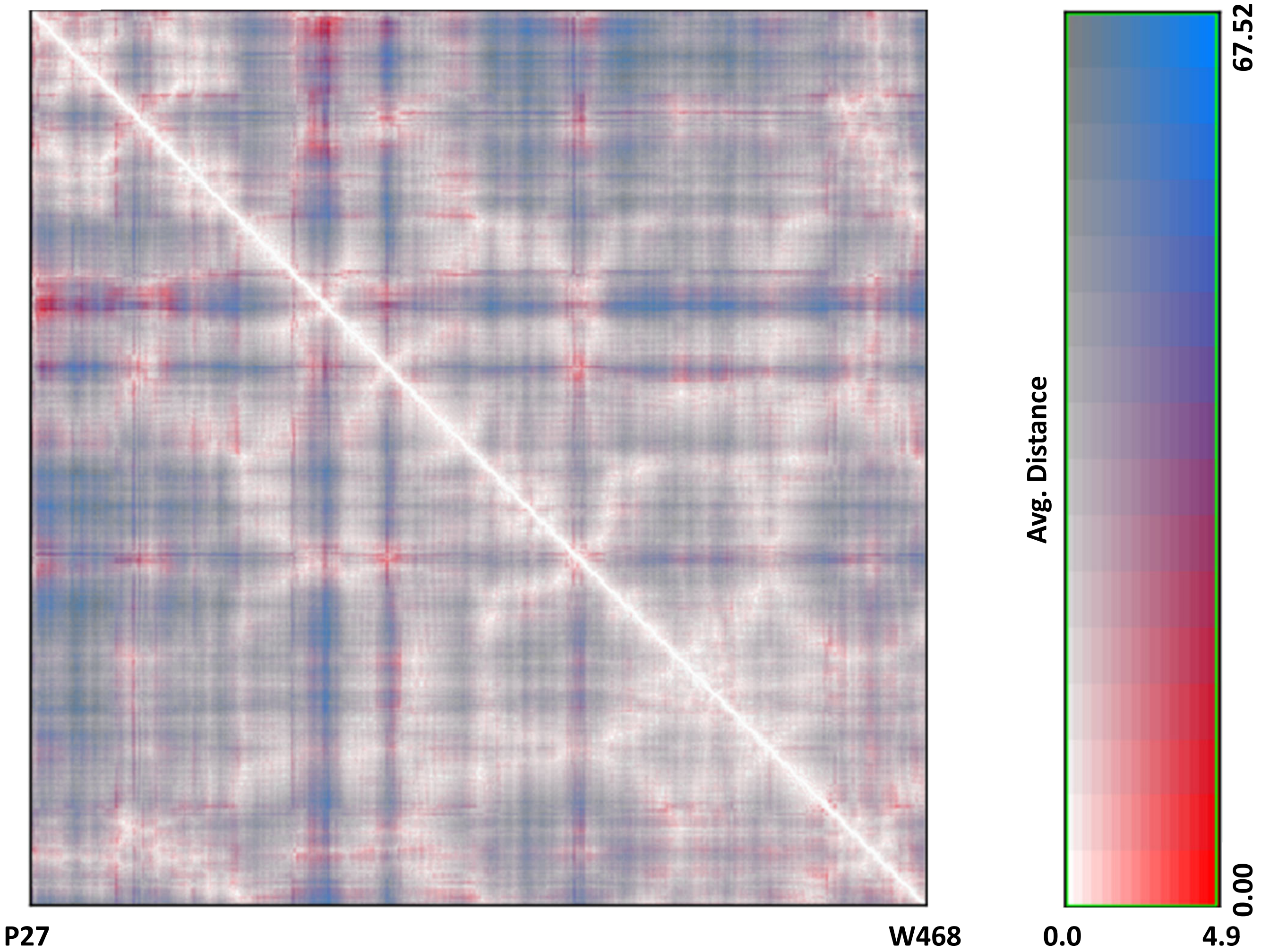

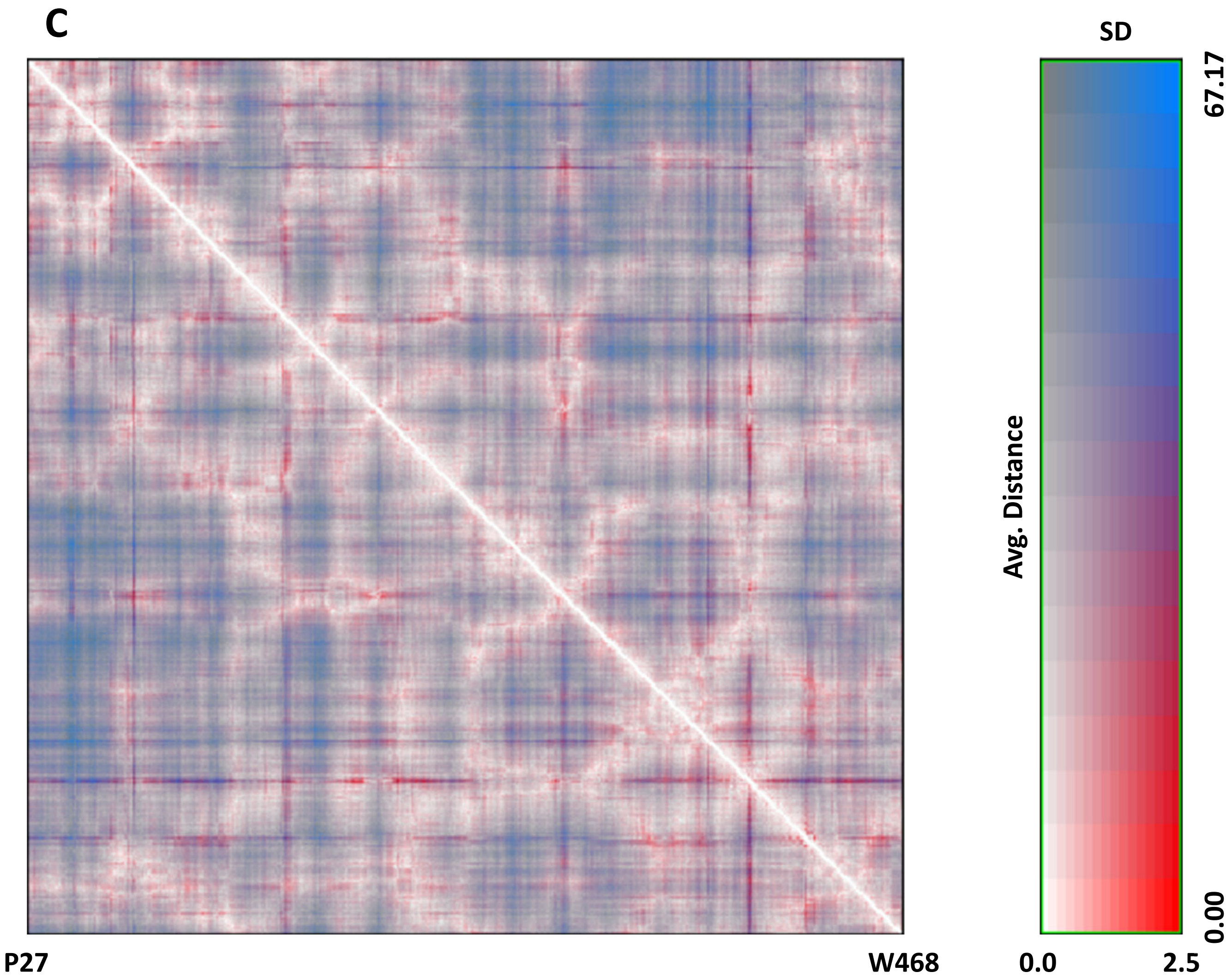
C
SD
67.17
Avg. Distance
0.00
P27
W468
0.0
2.5

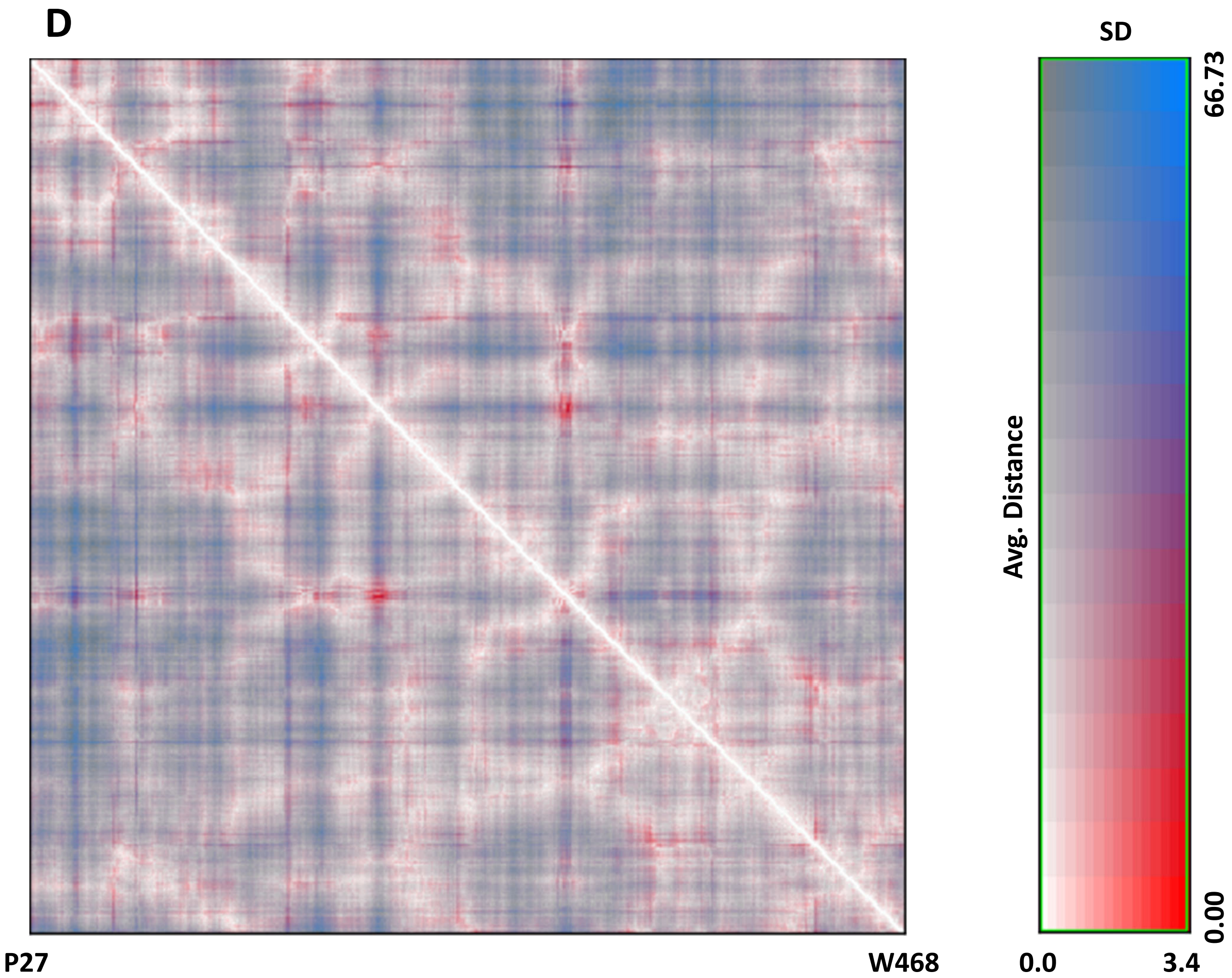
D
SD
66.73
Avg. Distance
0.00
P27
W468
0.0
3.4

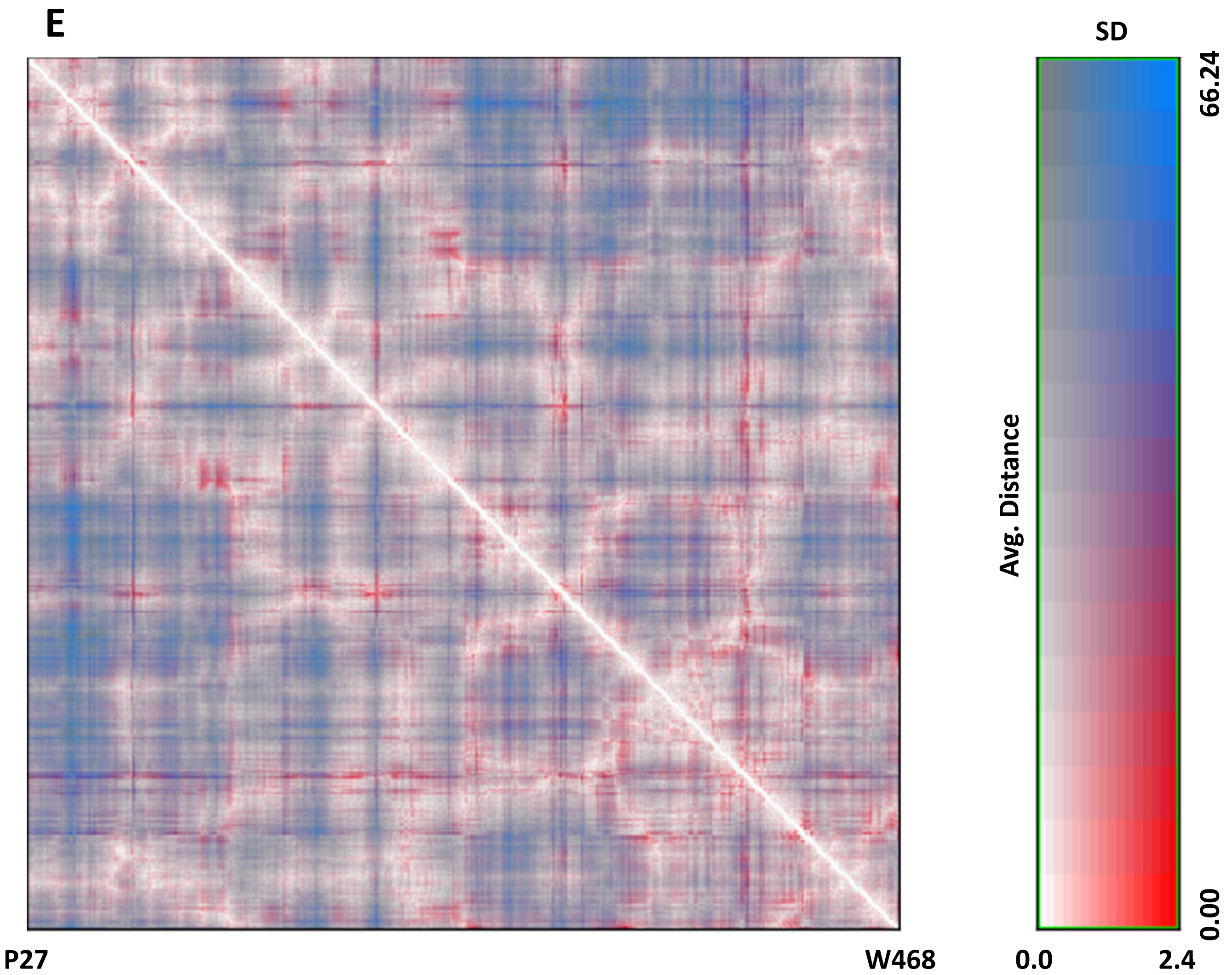

Supplementary Figure S3. Residue-Residue distance maps of Tyrp1 (A), C30R (B), H215Y (C), D308N (D), and R326H (E). Each structure had five PDB files aligned at timestamps of 0, 25, 50, 75, and 100 ns using the MatchMaker tool. Residue-residue distance maps were created to highlight structural changes over the duration of the MD simulation.

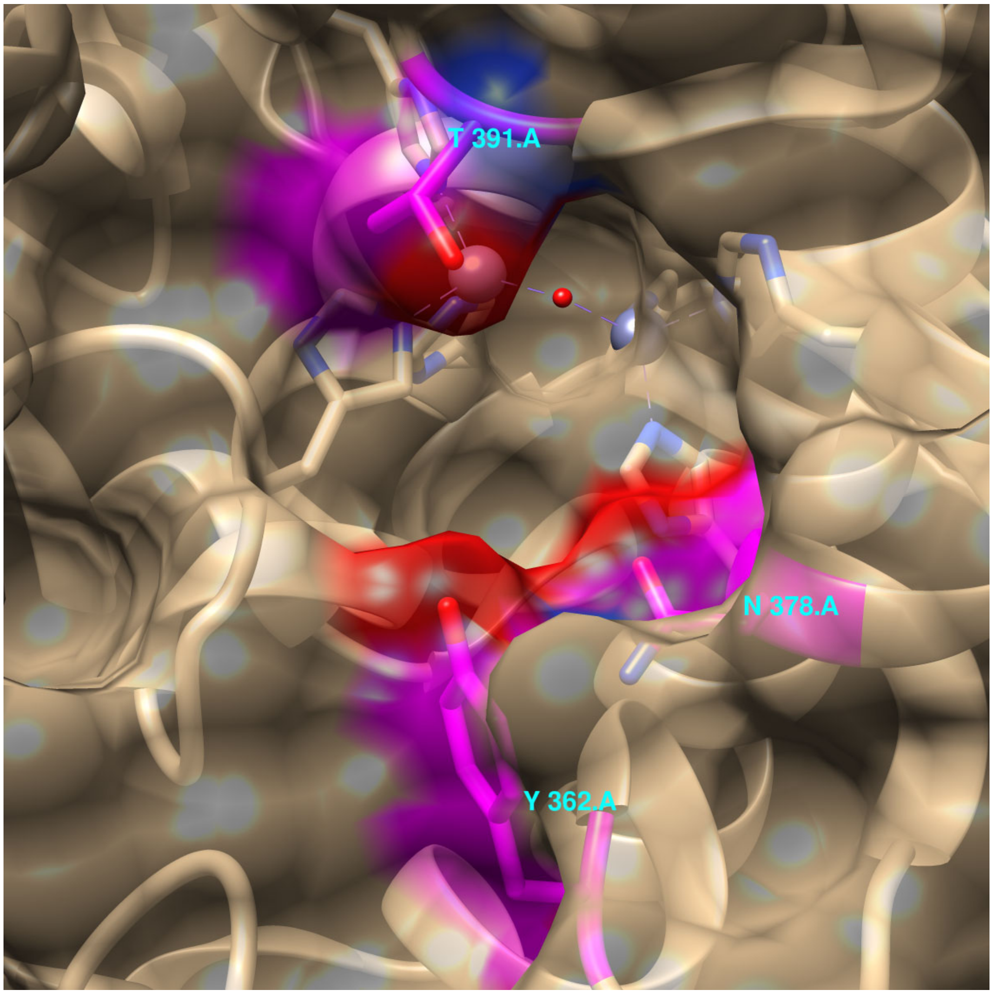

Supplementary Figure S4. Bottleneck residues of Tyrp1. The distances between T391 and both Y362/N378 form the narrowest portion of the entrance to the active site, and all three residues can form noncovalent interactions with DHICA. These distances were the order parameters used in the formation of the free energy landscape.
